# Text Detective: a rule-based system for gene annotation in biomedical texts

**DOI:** 10.1186/1471-2105-6-S1-S10

**Published:** 2005-05-24

**Authors:** Javier Tamames

**Affiliations:** 1 Alma Bioinformatics S.L., Ronda de Poniente 4, 28750 Tres Cantos (Madrid), Spain

## Abstract

**Background:**

The identification of mentions of gene or gene products in biomedical texts is a critical step in the development of text mining applications in biosciences. The complexity and ambiguity of gene nomenclature makes this a very difficult task.

**Methods:**

Here we present a novel approach based on a combination of carefully designed rules and several lexicons of biological concepts, implemented in the Text Detective system. Text Detective is able to normalize the results of gene mentions found by offering the appropriate database reference.

**Results:**

In BioCreAtIvE evaluation, Text Detective achieved results of 84% precision, 71% recall for task 1A, and 79% precision, 71% recall for mouse genes in task 1B.

## Introduction

Correctly identifying the entities and concepts that are mentioned in a text is a mandatory step for systems attempting accurate information retrieval and, especially, information extraction tasks. In the biomedical domain, important entities are genes and proteins, chemical compounds, drugs, diseases, pathways, etc. Adequate recognition of these concepts is often a difficult task, because biomedical nomenclature is often imprecise and ambiguous [1-3].

The worst case is undoubtedly that of gene name recognition. Gene names present the following difficulties for their detection:

• They can be mentioned as "full names" (such as "insulin" or "mitogen-activated protein kinase ") or symbols (abbreviations such as INS for "insulin" or MAPK for "mitogen-activated protein kinase". Symbols are often, but not always, acronyms). It is advisable to treat separately mentions of symbols and of full names, since the problems they present are different.

• Aliases: A given gene can have multiple names. For instance, the gene for the human fibroblast growth factor receptor has at least 11 different names. It is important to count on comprehensive lexicons or list of names. This is a problem both for symbols and for gene names.

• Homonyms: A given name can designate different genes. For instance, the name "PAP" can be associated with (is an alias of) at least five different human genes (pancreatitis-associated protein, development-differentiation enhancing factor 1, mitochondrial ribosomal protein S30, poly(A) polymerase alpha, and platelet growth factor associated protein 1). This creates an ambiguity problem, and external (expert) information on the characteristics of each of these genes is needed to disambiguate the mention. Since by definition a full name characterizes completely a gene mention, this is a problem only for symbols.

• Acronyms: A given name can stand for multiple meanings. For instance, the name "SCT", that stands for "secretin" gene, can also correspond to more than 20 other meanings, with "stem cell transplant" the most frequent one. For many genes, this impairs substantially the precision of the detection. This is a very serious problem affecting uniquely to symbols.

• Orthography: There may be small variations in the way of writing the gene names, such as the presence or absence of hyphens between words, as in "IL-2" compared with "IL2" or "IL 2". This can result in differences in the number of words of the gene name, which can impair the efficacy of the detection. This is a problem both for full names and symbols.

Also, for biomedical applications, it is often not enough to identify a gene mention in the text. It is also necessary to find a correspondence with some database identification for the gene, so that we can use its associated DNA sequence, for instance, thus eliminating any possible uncertainty about the identity of the gene. This constitutes a normalization step that is often ignored when discussing gene name recognition.

The factors above make gene name recognition a rather difficult task, that has been attempted mainly by three different approaches: Support vector machines [4-6], Hidden Markov models [7,8], and rule-based systems [9,10]. We have tried a rule-based approach, which allows us to encode a high amount of external knowledge (mainly compiled from databases) about the individual genes.

**Figure 1:**
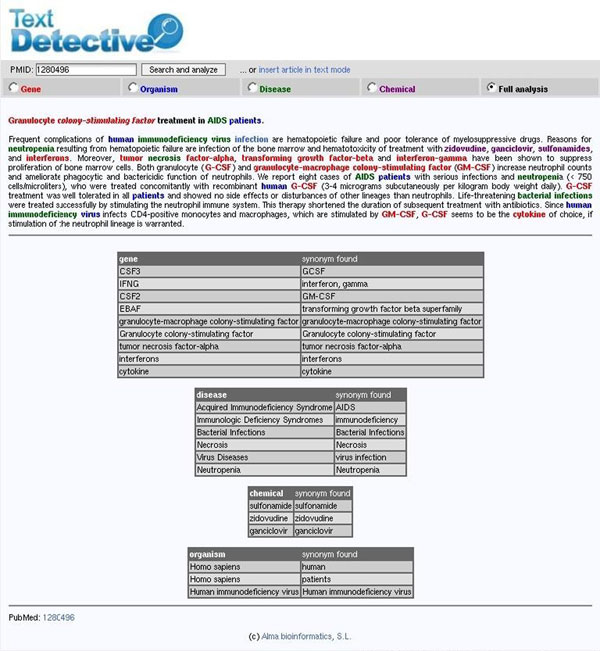
The system Text Detective is capable of annotating a wide range of biological entities, such as genes and proteins, diseases, chemical compounds, drugs, organisms etc.

The system we are presenting is named "Text Detective", and it is capable of annotating a wide range of biological entities, such as genes, proteins, chemical compounds, drugs, diseases, biological processes and pathways, etc (see figure 1). We will only discuss here the gene annotation machinery, and the performance of the system in BioCreAtIvE evaluation of automatic systems for gene annotation [11].

## Implementation

### Text tagging

As we have already mentioned, gene names can be found in two different ways: as full names (a functional description of the gene, such as "tumor necrosis factor" or "janus kinase"), and as gene symbols (an abbreviation or acronym, such as "TNF" or "JNK"). Since as we have discussed, the associated problems are different, we used different approaches for detecting each of the cases. But there are some initial steps common to both procedures.

The first common step is to parse (split in sentences, remove punctuation, etc) and tokenize the document. Then, every sentence in the document is processed independently.

Text Detective is then able to tag every word in the sentence according to biologically relevant categories. These categories are:

• CENTRAL: also known as "core terms", they are words that are very informative of the possible function of the protein associated to the gene. They are, for instance, words such as "kinase", "receptor", "transporter", etc. The presence of these words is (almost) always indicative of a gene name. They are tagged by a combination of rules (such as presence of suffixes such as "ase") and hand-crafted lexicons (list of words such as "receptor")

• CHEMICAL: These are chemical compounds, tagged by a set of rules (prefixes, suffixes and substrings) that follow carefully the chemical nomenclature. For instance, prefixes such as "hydroxy" or "bis", suffixes such as "one" or "amine", or substrings such as "chlor" or "oxo" can indicate that the word is part of a chemical name. A confidence level can be assigned according to the number of matched rules. Also a lexicon is used here, both to include chemical names that do not match the rules (with approximately 10000 entries, especially drug names such as "prozac" or "aspirin"), or to exclude non-chemical words that do match the rules (almost 9000 words, such as "chloroplast" or "examine"). Several sources of data were used, especially UMLS lexicon [12] and FDA listings of approved drugs (for instance, see [13])

• TYPE: Words such as "alpha", "a1", "c", "12", "TNF", that define the exact identity of the gene (distinguishing between "interferon alpha" and "interferon gamma", for instance). Words containing numbers, letters, greek letters, roman numerals, capital letters and combinations of these belong to this category. As a consequence, gene symbols (such as "TNFalpha") are also tagged as "type".

• LOCATION: Cellular, sub-cellular and tissue locations, such as "liver", "intestinal", "lymphocyte", "membrane", "mitochondrial", etc. They are tagged using a hand-crafted lexicon (containing more than 800 entries), starting from Gene Ontology [14] and UMLS [11] lexicons.

• BIOWORD: Biological relevant words that do not fall in the previous categories. We use a comparison of two different corpora (a biological one, and a non-biological one) for knowing if a word falls in this category. A word is a bioword if its frequency is much higher in the biological corpus than in the non-biological one. Since using this method there are some words loosely associated with biology (such as "flight", "method", etc) that can be recognized as biowords just by chance, the procedure is also refined using a Poisson statistics [15] to take into account clustering of words in the articles. The idea is that "relevant", content words, will be statistically clustered, that is, they will be cited in the same article more times than what will be expected by chance. The distribution of non-content words will show no clustering. In this way it is possible to select as biowords just content words over-represented in biological articles.

The rest of the words are tagged as "OTHER".

Notice that this is NOT a syntactic part-of-speech tagging, rather we try to recognize the role of the word in a possible gene mention.

An example of a tagged sentence follows:

Decay -> BIOWORD

accelerating -> BIOWORD

factor -> CENTRAL

(DAF) -> TYPE

is -> OTHER

a -> OTHER

complement -> BIOWORD

regulator -> CENTRAL

that -> OTHER

dissociates -> OTHER

autologous -> BIOWORD

C3 -> TYPE

convertases -> CENTRAL

which -> OTHER

assemble -> OTHER

on -> OTHER

self -> OTHER

cell -> LOCATION

surfaces -> BIOWORD

The tagging procedure can lead to some mistakes, especially in the "bioword" and "chemical" category (the first one because it is loosely defined, the second one because many rules and exceptions exist). Nevertheless, we estimate that errors are less than 10% in all categories.

Once all words are tagged, Text Detective attempts to discover gene mentions both as full names ("tumor necrosis factor alpha", "interleukin 1") or gene symbols ("TNFalpha", "IL 1"). The procedure is different for both instances.

## Identification of full names

In order to discover full names, the system extracts chains of words that can represent a gene mention. These chains are selected only if they fulfil several criteria. For instance, No "OTHER" words are allowed, and the presence of a "CENTRAL" word is needed. The resulting chains of words are possible gene mentions. For the example above, the possible gene mentions selected would be:

Mention 1: Decay accelerating factor DAF (Bioword, Bioword, Central, Type)

Mention 2: Complement regulator (Bioword, Central)

Mention 3: Autologous C3 convertases (Bioword, Type, Central)

In subsequent steps, these possible gene mentions are refined using filtering rules. For instance:

Rule 1: remove any plural at the end of a central word

Rule 2: remove isolated biowords at the beginning of a gene mention

Rule 3: remove mentions that comprise just one word.

The application of rule 1 turns mention 3 into "Autologous C3 convertase". Application of rule 2 turns mention 2 into "Regulator", and mention 3 into "C3 convertase". Application of rule 3 removes mention 2 ("regulator", just a single word).

## Identification of symbols

Gene symbols (TNF, EGR, p53) identification follows a slightly different procedure. As we said above, gene symbols are recognized as chains of "TYPE" words (one or more). But as we stated above, multiple meanings (acronyms) can exist for a given symbol, and therefore it is necessary to evaluate the context of the possible symbol in order to determine if such a mention it is indeed referring to a true gene.

Using a set of 940 articles annotated by experts, we have computed the probabilities of appearance of some words in the vicinity of gene mentions. These set of words conform a scoring matrix (Table 1), that is used to evaluate the context (the surrounding words) of a given symbol.

, Thus we have the following for the sentence:

"The function of c-fos gene in CC2 cells is partially inhibited"

The possible gene symbol "c-fos" scores 1.8 (word "function" at position -2) plus 5.0 (word "gene" at position +1), total 6.8, while the possible symbol "CC2" scores 0.5 (word "gene" at position -2) plus -5.0 (word "cell" at position -1), total -4.5.

The score for the symbol must exceed a minimum score in order to be declared a valid gene mention. This minimum score is set up independently for each possible symbol, by means of what we call "risk factor". Briefly, the risk factor is a number between 0 and 100 that indicates the probability that the symbol matches different meanings. In other words, it indicates the ambiguity of the symbol. As the symbol is more ambiguous, the risk factor is higher.

The risk factor is calculated combining several pieces of information. For a given symbol, all sentences in which it appears are extracted and word usage in these sentences is examined, comparing it with the word usage of sentences containing true genes. This is called "word usage information". Also, when the symbol is found between parentheses, it is very likely that the preceding words inform us about the meaning of the symbol (as in the sentence "we performed Stem Cell Transplant (SCT)."). In these cases, we look in these preceding words for terms that strongly indicate the presence of a gene, that is, we look for "CENTRAL" words. This is call "acronym information". Other criteria, such as the morphology of the symbol (presence of numbers, a capital letter at the end, etc.), can also be used. All these pieces of information are combined to obtain a single value for the risk factor, what in turn is used to modulate the minimum score needed. Thus, "SCT" is a symbol with high risk (word usage is often different from that of genes, and acronym information indicates that the symbol can match many meanings not related to genes), and therefore minimum score is set high. On the contrary, symbol "BRCA1" has a very low risk (word usage matches that of genes, and acronym information indicated that the symbol is always related to a instance of a gene), and therefore minimum score needed is very low.

**Figure 2:**
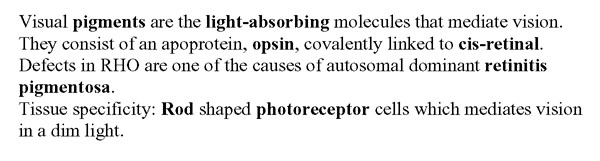
Example of a functional annotation extracted from SwissProt, for the gene of human rhodopsin. In bold letters, the keywords that can be extracted from this text. These keywords are selected because their frequency is low in the full corpus and also because they are relatively specific (They do not appear in more than 25 genes).

Additionally, if we have a list of gene names (that can be compiled from different databases, such as HUGO, SwissProt LocusLink, etc.), we could make use of the functional information available for a given gene. It is possible to extract some terms that are informative and specific for the function of a given gene (Figure 2). For selecting these terms, the criteria followed is that they have low frequency in the full corpus and are not present for many genes. The final result is a set of keywords for each gene. Now, if the article is mentioning a given gene, the text will be scanned looking for its particular keywords. If they are found, they will add a score based on the informative content of the keyword. This additional score will complement that obtained by means of context evaluation.

After this step, we will have a set of possible gene names mentioned in the article as gene symbols. This result is combined with the identification of full names to yield the final set of genes annotated for the article.

## Matching with a list of gene names

**Figure 3:**
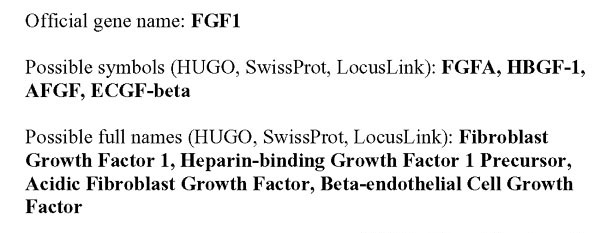
A single entry for a gene in the lexicon. Information is compiled from different databases.

In order to identify the exact gene reference, we must match the gene mention we have found (full names or gene symbols) with a list (lexicon) of possible genes (full names or gene symbols), that can be extracted from different databases (HUGO, MGI, SGD, SwissProt, etc.). An example of such a lexicon can be found in figure 3. This allows the complete identification of a gene mention, so that it is possible, for instance, to retrieve the associated DNA sequence. This can be seen as a normalization step.

Again, the procedure that Text Detective uses is different for full names and for gene symbols. For full names, we have tagged the lexicon, the list of full names, using the same procedures described above. For instance, the lexicon entry "gamma-aminobutiric acid receptor delta" is tagged as:

gamma-aminobutiric -> CHEMICAL

acid -> CHEMICAL

receptor -> CENTRAL

delta -> TYPE

This is done for all the entries in the lexicon.

A match between the full name found in the article and an entry in the lexicon is only scored if several criteria are fulfilled. For instance, all central terms and chemical compounds must be present both in the full name found in the article and the full name in the lexicon. All types must be also present. This matching procedure is very flexible, so that word ordering, dashes, slashes, brackets, etc, do not influence the result. A match is only scored if just one gene in the list fulfil all criteria, and then the "official" reference is returned. In case several genes in the list could match the gene mention, ambiguity is detected and no identification is provided.

For instance, suppose that in the article we are analysing, we have found a mention of "dopamine D3 receptor". In our lexicon compiled from HUGO database of human genes, the closest entry refers to gene DRD3, with the full name "Dopamine D3 receptor precursor". In this case, the mention and the possible match have in common all chemicals ("dopamine"), all central words ("receptor") and all types ("D3"). The only word not in common is a bioword ("precursor"). Therefore, we score a match and identify the mention of "Dopamine D3 receptor" with DRD3.

Now suppose that the article mentions "GDNF neurotrophic receptor alpha". The lexicon has three possible entries matching this mention. They are: GFRA1 ("GDNF family receptor neurotrophic factor alpha 1"), GFRA2 ("GDNF family receptor neurotrophic factor alpha 2"), and GFRA3 ("GDNF family receptor neurotrophic factor alpha 3"). In this case, some types are unmatched between any of the three possible entries and the gene mention found (the types "1", "2" and "3"). Therefore it is not possible to choose any of the three, and the mention to "GDNF neurotrophic receptor alpha" remains unmatched.

For gene symbols, we try to match all possible gene symbols found in the article with a lexicon of allowed gene symbols, extracted from different databases (HUGO, LocusLink, MGI, SGD, SwissProt, etc.). As before, the matching procedure is flexible. But ambiguity can be present and a given symbol can stand for different genes (for instance, gene symbol "PAP" in humans can stand for five different genes)

To overcome the ambiguity, we use the keywords extracted for every possible gene, as was explained in the previous section. In case that several possibilities are available, the one with more keyword is selected (Table 2).

Finally, the system assembles the information about full names and gene symbols in one single result, and returns it as the final output.

## Results

The performance of our method was evaluated in our participation in BioCreAtIvE challenge. To validate the results, the measures of precision and recall were used, defined as:



where TP is the number of true positives (the annotations made that are correct), FP the number of false positives (the annotations made that are not correct), and FN the number of false negatives (the genes missed).

**Figure 4:**
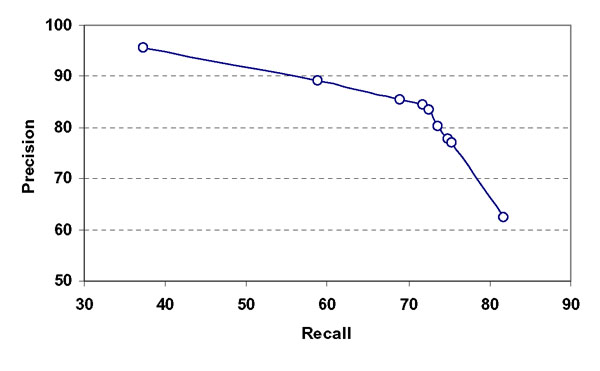
Precision/recall plot for gene identification. Results from BioCreAtIvE task 1A data set.

Just for the gene identification process (BioCreAtIvE task 1A), the system achieves an average of 84.2% precision, 71.7% recall. Figure 4 shows the results at different levels of precision and recall. As expected, a trade-off between precision and recall exists that can be tuned mainly by modifying the parameters influencing the minimum score required for accepting a gene mention. Both parts of the gene identification process (detection of full names and detection of symbols) achieve very similar results, indicating that the system is very well balanced. These results are similar to the ones we obtain by annotating a set of hand-curated articles of our own (500 articles), but precision and recall is higher in our tests, mainly due to the different criteria of "what-is-a-gene" and the very limited context information provided in BioCreAtIvE evaluation (see discussion section below).

**Figure 5:**
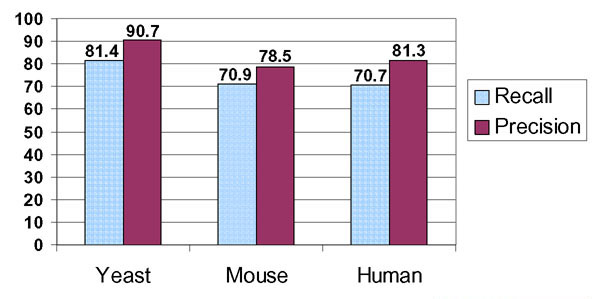
Performance of complete system (including matching with gene lexicons) for different organisms. Data for yeast and mouse come from BioCreAtIvE evaluation (task 1B), data for human come from a hand-annotated set of 500 articles.

For estimating the performance of the system including the matching against lists of gene names (BioCreAtIvE task 1B), we decided to work only with mouse and yeast, since names for fly needed of some adaptations to the system (some of them, like "sevenless", were not recognized as symbols using the described tagging rules). Also, detection of human genes has been attempted in our own tests, based on 500 articles manually annotated by human experts. The results can be found in figure 5.

## Discussion

Text Detective is a rule-based system for annotating and normalizing gene mentions in texts, that reaches high precision and recall for this task. Nevertheless, there are several points that must be clarified.

First at all, it is difficult to reach a consensus of what should be annotated as a gene and what should not. For example, some users want to consider as gene mentions instances such as regions or motifs ("HindIII fragments", "HLH motif", "silent mating type loci"), promoters ("Oct3 promoter"), etc. That was the case in BioCreAtIvE task 1A evaluation. In our view, these instances are not genes and the system is not tuned to detect them. Therefore, the performance of the system may be different depending on what the user is expecting. This illustrates also the difficulties in creating Gold Standards for automatic evaluation of systems dealing with gene name recognition.

The performance of the system is dependent on the length of the text provided. This is because in order to evaluate a gene/protein mention, the system takes into account all mentions of the possible gene/protein in the text. The context of all the instances is evaluated, and global features are extracted. That means that the results improve as the text grows, since more information can be used. Optimal results are achieved when using a complete PubMed abstract (usually between 10–15 sentences). This impairs the results of the system in BioCreAtIvE task 1A, where just one sentence out of context was provided. In fact, most of the false negatives occur when no context information could be found.

There are also many instances of difficult cases, in which the citation to the gene is obscure or complex. For instance, we found an article where the notation "lpa(1-3)" was used to refer to lpa1, lpa2 and lpa3. These instances present a real challenge for an automatic system, and very sophisticated rules must be devised to deal with them. We find that this is the case for 20–25% of our false negatives in our participation in BioCreAtIvE task 1A.

In the normalization step, the system is highly dependent of the quality of the lexicon (the gene list) provided. In our experience, these lists require careful examination and filtering to remove some inconsistent annotations. Also, as the system relies on keywords extracted from functional annotations to perform the normalization step, there is a compromise between the amount of text provided and the quality and accuracy of it.

Finally, we want to raise our concerns about the measures used for evaluating the performance of this and similar systems. We have observed that the precision and recall of the system is not uniformly distributed for all genes. In other words, 80% precision does not mean that this and similar systems make just one error out of five annotations for every gene. Instead, we have observed that errors concentrate in few, very difficult cases. For instance, when the gene symbol is a common acronym for many other meanings (SCT could be the case). Therefore, the scenario is closer to one in which the systems annotate completely right 80% of the genes, and fail often for the 20% remnant. If this is true, we may be further from the solution of the problem than it seems.

## Figures and Tables

**Table 1:** Some entries in the context matrix. Scores are derived from the ratio between frequency of the given word in the given position with respect to the gene (position +1 means immediately after the gene name, position +2 means two words behind, etc.) and the total frequency of the word.

WORD	POSITION					
	-3	-2	-1	+1	+2	+3
gene	0	0.5	5.0	5.0	0.5	0
function	0	1.8	0	2.1	0	0
cell	0	0	-2.5	-5.0	0	0

**Table 2:** Example of disambiguation using keywords. The symbol ERK can match two different genes: EPHB2 and MAPK1. Analysing the article, no keywords can be found for EPHB2, but keywords "microtubule-associated" and "map2" are found. Therefore, the system assumes that the real identity of ERK is MAPK1.

Symbol found	Official name	Keywords
ERK	EPHB2	
	MAPK1	microtubule-associated; map2
